# Postoperative dizziness after cochlear implant surgery: can it be caused by air?

**DOI:** 10.3389/fneur.2024.1520472

**Published:** 2024-12-23

**Authors:** Manuel Christoph Ketterer, Friederike Everad, Niklas Lützen, Ann-Kathrin Rauch, Antje Aschendorff, Susan Arndt, Till F. Jakob

**Affiliations:** ^1^Department of Otorhinolaryngology, Medical Center – University of Freiburg, Faculty of Medicine, University of Freiburg, Freiburg, Germany; ^2^Department of Neuroradiology, Medical Center – University of Freiburg, Faculty of Medicine, University of Freiburg, Freiburg, Germany

**Keywords:** cochlear implant, Vertigo, Pneumolabyrinth, dizziness, surgery, hearing loss

## Abstract

**Objectives:**

Multiple studies have described the onset and variable incidence of postoperative acute vertigo following cochlear implant (CI) surgery. However, postoperative imaging has not yet been specifically evaluated with special focus on vertigo. The aim of this study is to assess the incidence and causes of new-onset, acute postoperative vertigo following CI surgery using cone beam computed tomography (CBCT).

**Materials and methods:**

This is a retrospective study involving ten patients who experienced postoperative dizziness and ten matched controls without dizziness. All patients received a cochlear implant (CI) between 2020 and 2024. The matching analysis was performed based on the implant, electrode array, and access to the cochlear. We analyzed the postoperative CBCT scans for changes suspicious of air trapping, a so-called pneumolabyrinth in the vestibule using minimal Hounsfield Units (HU).

**Results:**

We compared postoperative CBCT images for electrode array position monitoring in ten patients with vertigo versus ten patients without vertigo after CI surgery. Among the ten patients with postoperative dizziness, six showed suspicious changes in the vestibule consistent with the presence of air. These air-related changes were observed in the vestibule and, in one patient, additionally in the horizontal semicircular canal. Minimal HU were significantly different and confirmed the suspicion of intravestibular air.

**Conclusion:**

This is the first study to describe the suspicion of intravestibular air in CI patients with postoperative vertigo. Therefore, suctioning after the fenestration of the round window membrane or the endosteum after cochleostomy, as well as actions such as bending, pressing, and nose-blowing by the patient, should be strictly avoided. Furthermore, this finding highlights the importance of carefully sealing the electrode array at the cochleostomy site with connective tissue. Risk factors for the development of a pneumolabyrinth with air in the vestibule include intralabyrinthine or intracranial pressure changes, large cochleostomies or a second cochleostomy and electrode placement in the scala tympani.

## Introduction

The occurrence of acute postoperative vertigo is recognized following Cochlear Implantation (CI) and has been reported with variable incidence. Studies have reported postoperative dizziness rates as up to 60% (1–5). Some studies have suggested that membrane damage of the sacculus is associated with a loss of function in cervical vestibular evoked myogenic potentials (cVEMPs) and with acute postoperative vertigo (1, 6, 7). In their meta-analysis, Hänsel et al. (8), reported new-onset postoperative vertigo in 202 out of 1743 patients (17.4%) following CI surger. Martin et al. (9), 16.9% postoperative vertigo in 71 included CI patients, along with associated risk factors such as straight lateral wall electrode arrays and surgical trauma. They evaluated cochlear coverage and scalar position via cone beam computed tomography (CBCT) and found no significant difference in postoperative vertigo for either cochlear coverage or scalar position.

Previous studies have described a lower occurrence of acute postoperative vertigo in patients with round window-inserted CIs, but these findings were based on small study cohorts (8, 10, 11). Karimi et al. (12), reported acute postoperative vertigo in patients with possible perilymphatic leakage and observed the disappearance of postoperative dizziness following the resealing of the inserted electrode array. Nevertheless, postoperative imaging of the vestibule has not yet been evaluated in terms of acute postoperative vertigo.

The aim of this study is to assess on air suspicious changes in the vestibule by CBCT in acute postoperative vertigo following CI surgery. The occurrence of air in the labyrinth, the so called pneumolabyrinth, is described amongst others following trauma with temporal bone fracture (13), barotrauma (14, 15), Eustachian tube air inflation (16), and stapes surgery (17). According to a review by Botti et al. (18), the most common site of air entrapment is the vestibule, followed by the cochlea and the most common causes were head trauma with temporal bone fracture followed by stapes surgery. Clinical symptoms of a pneumolabyrinth are hearing loss and vestibular symptoms (18). The pneumolabyrinth directly after CI surgery or in the course can be another cause, so far this cause has mainly been described in case reports (19–25). The pneumolabyrinth can be diagnosed by CT or CBCT scans. In this case, air appears hypodense on imaging. The radiodensity is described in Hounsfield units (HU). Water has an attenuation of 0 HU, whereas air has typically an HU number of −1,000 (26).

## Methods

We conducted a retrospective study on CI patients enrolled between 2020 and 2024. Ten patients who reported dizziness during hospitalization after CI surgery were included in the study and one matched control without dizziness for each vertigo patient, so that a total 20 patients were included. The matching was performed with regard to the implant, electrode array, cochlear access and age. Twelve patients underwent imaging through cone beam computed tomography (CBCT) and eight patients high resolution computed tomography (HRCT) before surgery.

**Figure 1:**
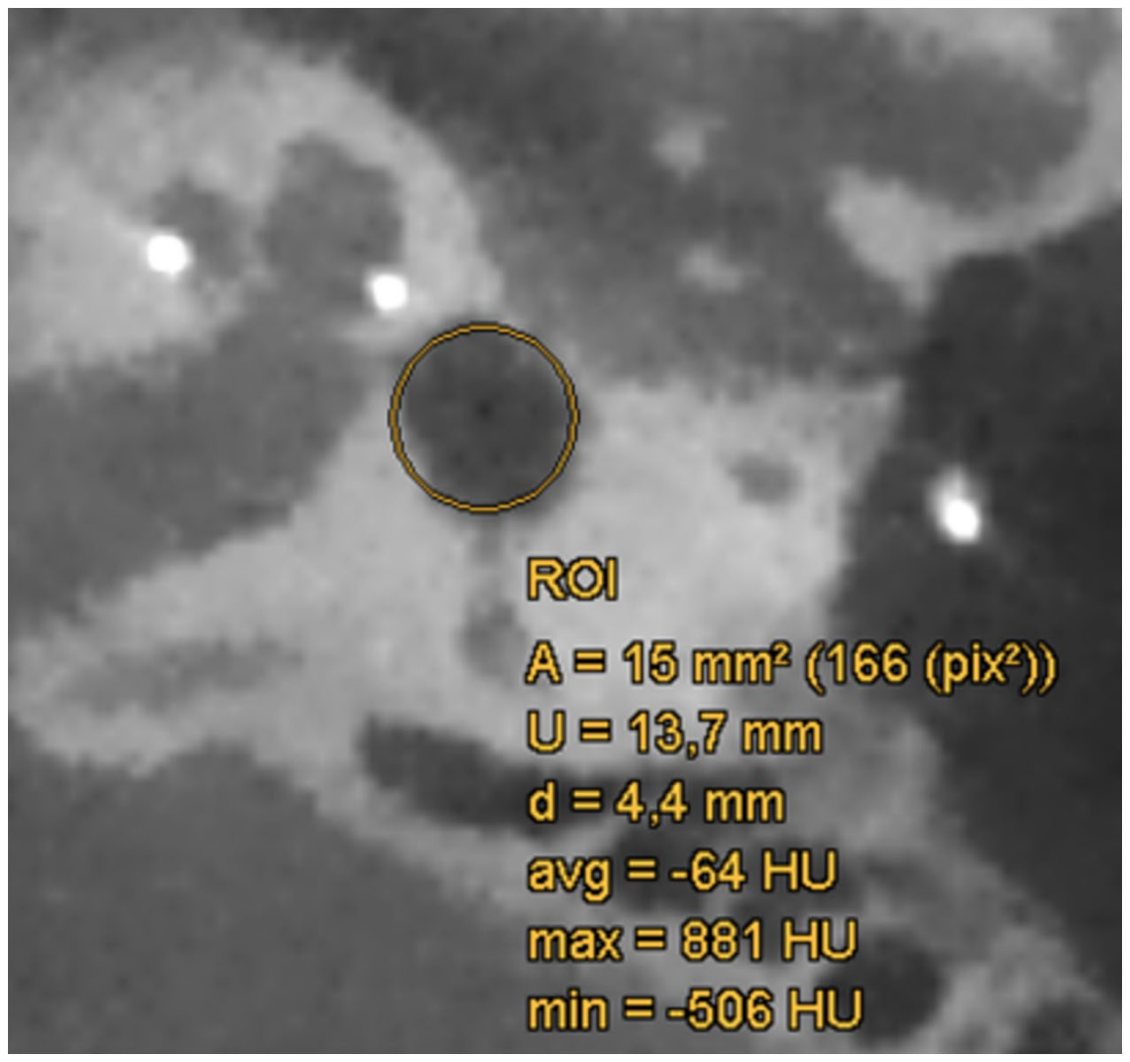
CBCT scan of the labyrinth postoperatively after CI surgery with region of interest (ROI; yellow circle) in the area of the vestibule. The diameter (d) of the ROI is 4.4 mm, the minimal Hounsfield unit (HU) is at - 506 HU.

Postoperatively, we performed CBCT (NewTom 5G, Cefla s.c., Imola, Italy) one to three days after surgery in all patients. All pre- and postoperative CBCT scans were evaluated by two experienced CI surgeons and a neuroradiologist using the DeepUnity Diagnost 1.2.0.3 program (DH Healthcare GmbH, Germany). The minimal Hounsfield units (HU) in the vestibule in the area of the suspected air were noted. The sectional plane was chosen so that the part of the labyrinth where the cochlea, the lateral semicircular canal and the vestibule are sliced is shown. A region of interest (ROI) with a diameter of 4–5 mm was then placed in the vestibule and the minimal HU was noted (Figure 1). We performed a video head impulse test (vHIT) preoperatively. Postoperatively, we conducted vHIT 4 to 6 weeks following CI surgery in the matching cohort and in patients with vertigo.

Statistical analysis was done with Prism 7 software (GraphPad Software, Inc., La Jolla, CA, USA). We used the nonparametric Kruskal–Wallis test for statistical analysis. The significance level was set to *p* < 0.05. Significances were differentiated into (***) for *p* < 0.001, (**) for *p* < 0.01, and (*) for *p* < 0.05.We received ethical approval from the Hospital’s Ethics Committee following the Declaration of Helsinki guidelines (Washington, 2002) (Ethics Committee approval number: 129/19; amendment 240,022) and registered this study in the German Clinical Trials Register (www.drks.de / DRKS00034647).

## Results

All patients received a unilateral cochlear implant and met the indication criteria. The average age of the patients was 61.3 years (19 to 76 years) in the vertigo group and 54.9 years (21 to 72 years) in the control group (Tab. 1). Patients were free to choose the CI manufacturer. Middle ear access was achieved after mastoidectomy via a posterior tympanotomy. One patient had a radical cavity (patient B) and in two patients an intracochlear schwannoma was removed during CI surgery by a second cochleostomy in the second turn of the cochlea (patients E and F). Electrode insertion was performed via the round window in 14 patients and via cochleostomy in six patients (Table 1). In all patients, following the electrode insertion, the round window or cochleostomy was sealed with connective tissue.

**Table 1:** Characteristics of the six patients with changes in the vestibule suspicious for air (A-F), four patients with vertigo without air in the vestibule and 10 controls.

Patient	A	B	C	D	E	F	Vertigo without air (*n* = 4)	Controls without vertigo (*n* = 10)
Age in years	63	68	72	66	41	66	59.6 (19–76)	54.9 (21–72)
Side of implantation	Left	Rigth	Left	Left	Left	Left	3 left, 1 right	6 left, 4 right
Implant	Cochlear 622	AB MS	AB Slim J	Cochlear 612	MedEl Flex 26	MedEl Flex 28	2 Cochlear 622 1 MedEl Flex 26 and 28	Matched to vertigo group
Approach	RW	RW	RW	CS	Ext. RW + 2^nd^ CS apical	CS+ 2^nd^ CS apical	RW or CS	Matched to vertigo group
Scalar position	ST	SD 180°	ST	SV	ST	ST	3 ST 1 SD apical	8 ST 1 SD 178.5° 1 SV
Nystagmus	Yes, grade 2 rigth	no	Yes grade 1 left	No	No	Yes at day 3, grade 1 rigth	In 2 patients	No
Minimal HU	−506	−125	−244	−419	−269	−448	115.5 ± 58.9	126.6 ± 93.7
Location of air	Vestibule, HSC, basal Cochlea	Vestibule	Vestibule	Vestibule, basal and apical cochlea	Vestibule, apical cochlea	Vestiblule, apical cochlea	No air	No air
Specifics	EVA	Radical cavity	-	Incomplete partition II	Excision of ICS	Excision of ICS	–	–

HU, hounsfield Unit; RW, round window; CS, cochleostomy; ST, scala tympani; SD, scalar dislocation from tympani to vestibuli; SV, scala vestibuli; HSC, horizontal semicircular canal; EVA, enlarged vestibular aqueduct; ICS, intracochlear schwannoma.

**Figure 2:**
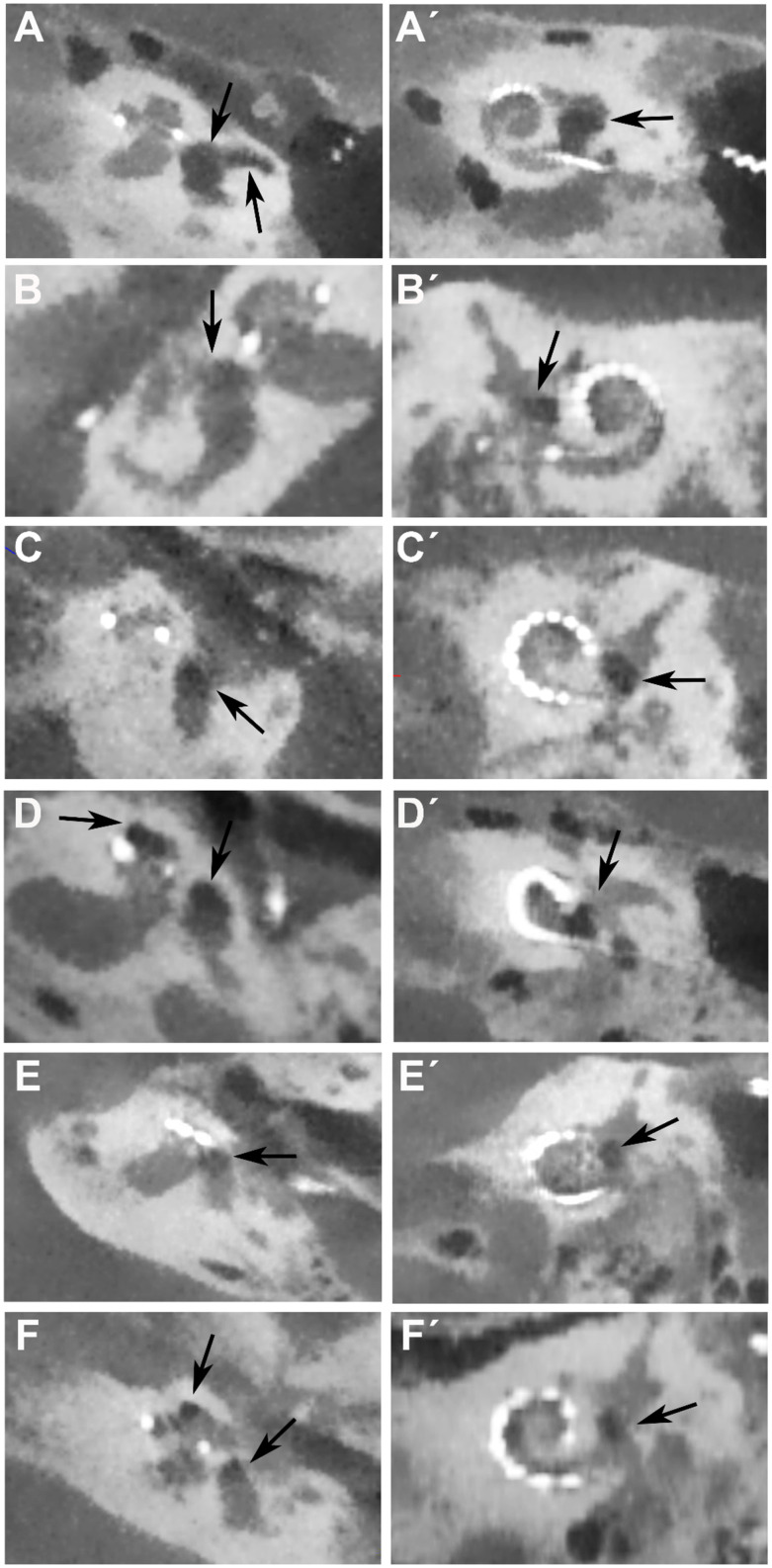
Postoperative CBCT images for electrode position control in cochlea view of the six patients **(A–F)** with vertigo and on air suspected changes in the vestibulum after CI surgery. Arrow shows localization of air in the vestibule in the axial view **(A–F)** and coronal view **(A´-F´)**. In patient **(A)** there is additional air in the lateral semicircular canal and in patients **(D,F)** additional air is found in the apical turn of the cochlea (second arrows).

We compared postoperative CBCT images for electrode position monitoring of ten patients who suffered from vertigo after CI surgery with ten patients who had no vertigo after CI surgery. In the CBCT scans, attention was focused on suspicious changes in the vestibule, the semicircular canals, and the cochlea. Of the ten patients who had dizziness post-operatively, six patients had suspicious changes in the vestibule on air (see Figure 2 and Table 1). The changes suspicious for air were observed in the vestibule and, in one patient (A), additionally in the horizontal semicircular canal and the cochlea (Figures 2A,A´). The clearest indication of air was observed in patient A of all six patients who showed suspicious changes in the vestibule. Post-operatively, the patient did not experience any dizziness. However, after bending down, sudden dizziness with nystagmus occurred. Additionally, the patient had an enlarged vestibular aqueduct (EVA), but during the cochlear implant surgery, there was no gusher (Table 1). In a revision surgery, which was performed after 2 days, there was no leakage of fluid in the area of the round window. Nevertheless, the area was sealed again and the dizziness improved over time.

In three patients additional air was seen in the cochlea (patients D, E, F; Figure 2), in two of these patients (E and F) a second cochleostomy was performed in the apical turn of the cochlea and an intracochlear schwannoma was removed. The second cochleostomy was also sealed by connective tissue.

Air was also found intracochlearly in patient D. In this patient, a cochlear abnormality of an incomplete partition II was found and therefore a bigger cochleostomy was performed.

The scalar electrode position was mostly the scala tympani (ST). In the vertigo group with air in four patients the electrode was placed in the ST (patients A, C, E, and F; Table 1), in one patient there was a scalar dislocation (SD) from the ST to the SV (scala vestibuli) at 180° (patient B). In one Patient (D) the electrode was in the SV. In the vertigo group without air in three patients the electrode was located in the ST and in one patient an apical SD was found (Table 1). In the control group, the electrodes were in the ST in eight cases, in the SV in one case and in one case there was a dislocation from ST to SV at 178.5° (Table 1).

In three patients in the group with suspicious changes of air postoperatively a preoperative CBCT did not show any suspicious changes in the vestibule and the minimal HU ranged from −12 to 94. In the other three patients in that group HRCT was performed preoperatively so that it was not possible compare the pre- and postoperative scans using the HUs, but no air was visualized.

The matched study patients did not demonstrate suspicious differences in vHIT or VNG preoperatively versus postoperatively. Patients with vertigo and pneumolabyrinth did not demonstrate highly suspicious vHIT postoperatively, except patient A, where air was also found in the ipsilateral horizontal semicircular canal and in the vestibule (Table 2).

**Table 2:** video assisted head impuls test (vHIT) preoperatively and postoperatively of patients A to F [p = posterior, l = lateral (=horizontal), a = anterior semicircular canal].

Patient	vHIT (preop.)		vHIT (postop.)	
	Saccades	Gain	Saccades	Gain
A	No	p: 0.83 l: 0.80 a: 0.83	Yes	p: 0.49 l: 0.29 a: 0.74
B	No	p: 0.79 l: 0.73 a: 0.73	Not tested	Not tested
C	No	p: 0.71 l: 0.82 a: 0.87	No	p: 1.01 l: 0.96 a: 0.84
D	No	p: 1.35 l: 0.99 a: 1.12	No	p: 0.89 l: 1.46 a: 1.07
E	No	p: 0.67 l: 0.82 a: 0.79	No	p: 0.66 l: 0.82 a: 0.80
F	No	p: 0.86 l: 0.54 a: 0.75	No	p: 1.21 l: 0.76 a: 0.80

**Figure 3:**
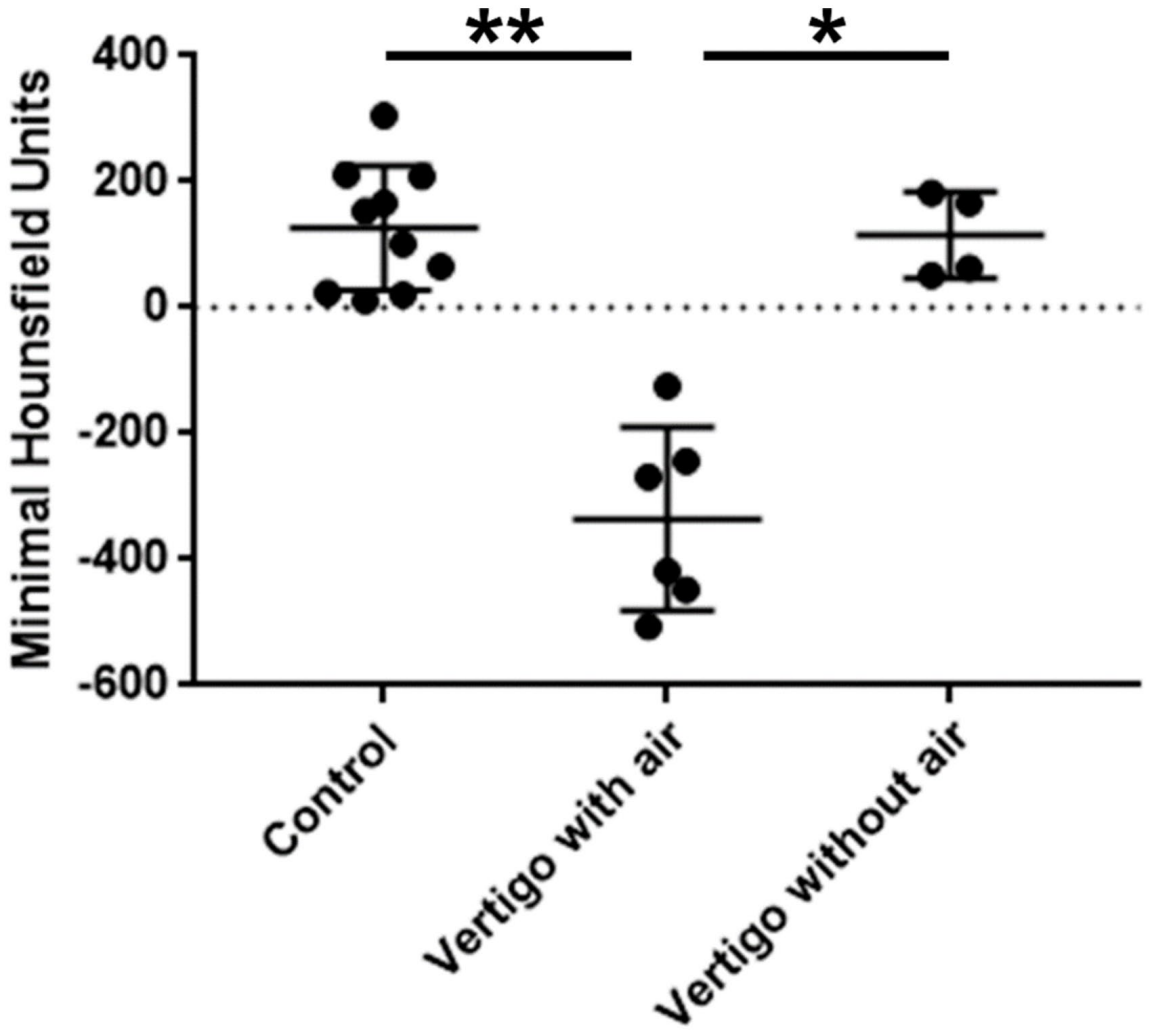
Comparison of the minimal Hounsfield units between the groups vertigo with air, vertigo without air and control. Significant differences were found between the vertigo with air and vertigo without air group and the control group. There was no difference between the vertigo without air group and the control group.

Minimal HUs ranged from −125 to −506 (mean − 335.2 ± 132.8) in the vertigo-group with changes in the vestibule and from 51 to 182 (mean 115.5 ± 58.9) in the vertigo-group without changes in the vestibule (Table 1 and Figure 3). In the control group (patients with no vertigo post-operatively) minimal HU ranged from 11 to 305 (mean 126.6 ± 93.7; Table 1 and Figure 3). There was a significant difference in the minimal HU between the patients with CBCT scans suspicious for air in the vestibule and those with dizziness but not such changes in the vestibule (*p* = 0.029; Figure 3). In addition, a significant difference between the patients with suspicious changes and controls was observed (*p* = 0.003; Figure 3). However, such a difference was not found in patients suffering from dizziness but without changes in the vestibule and controls (*p* > 0.999; Figure 3).

## Discussion

Following numerous studies that examined the incidence of acute postoperative dizziness following cochlear implant (CI) surgery and described a wide range of its occurrence (1–5), this study focused on identifying abnormalities in postoperative dizziness in patients through CBCT. A total of ten patients with acute postoperative dizziness were investigated and matched with ten CI patients each who did not exhibit dizziness. To our knowledge, there is only one study to date that focused on pneumolabyrinth after CI surgery. Here, however, air was mainly found in the cochlea of every implanted ear and only in one of 53 patients in the vestibule. The volume of the pneumolabyrinth in the cochlea was significant larger in patients with postoperative dizziness (27).

Although several studies (10, 11) have described cochleostomy as a risk factor for acute postoperative dizziness, our cohort of ten patients with dizziness included four patients who underwent cochleostomy and six patients who had round window insertions. Since most of our CI patients are implanted via the round window and therefore the number of these patients is significantly larger, the ratio of 6:4 could suggest the assumption that a cochleostomy is associated with an increased risk of postoperative vertigo (Table 1).

In six of the ten patients with dizziness who were suitable for analysis, we identified suspicious changes in the vestibule, which appeared to be indicative of air on HU, despite all patients being sealed with connective tissue post-insertion. The fact that the minimal HUs are not around −1,000 but on average around −335 HUs can be explained by the small volume of the vestibule and the resulting possible artifact overlays. Thus, the pathway by which the air entered the vestibule remains to be discussed. The long route via the cochlea from the ST to the SV and then to the vestibule appears unlikely. Other possibilities include passage through the scala media and then the ductus reuniens, potentially due to injuries to the basilar membrane during implantation or through existing fissures or diffusion, followed by pressure increases, such as when blowing the nose or bending over, as described in patient A. The pathophysiology of a pneumolabyrinth discussed in literature are external forces on the oval or round window, fistula ante fenestram, Hyrtl’s fissure, and micofissures that allow air entering into the perilymphatic space (18). In general, pressure changes or large accesses into the cochlea appear to be associated with an increased risk of a pneumolabyrinth. In the existing case reports on a pneumolabyrinth, the patients had a ventriculoperitoneal (VP) shunt which partially did not function (19, 22, 23), an enlarged vestibular aqueduct syndrome (EVA) (24, 25) or a large cochleostomy due to the electrode size (20). In one case, an electrode position in the SV was suspected, but the CT image in the publication also seems to show an EVA (21). In five of our six patients with air in the vestibule, there were also abnormalities in the sense of an EVA (patient A) or large or a second accesses to the cochlea (patient D large cochleostomy for malformation of the cochlea and patients E and F second cochleostomy in the apical turn for removal of intracochlear schwannoma). Patient D exhibited an incomplete partition II with an enlarged cochleostomie. In patient B a scalar dislocation at 180° was found and in patient D the electrode position was in the SV. Only in patient C no anomalies were found. It can be concluded from this that the risk of a pneumolabyrinth is very low in the case of “normal” anatomy and insertion of the electrode via the round window without electrode dislocation.

Some studies have shown that CI surgery leads to shifts of the amplitude in the VEMPs, but a clear cause for this is not yet known (for review see (8, 28)). One possible explanation for the observed changes in cVEMPs could be the air inclusions we have identified in the vestibule. Since we did not examine VEMPs, this would be interesting for a follow-up study. Especially in cases with preoperative abnormalities such as EVA, intracochlear schwannomas, or cochlear malformations, this examination could be conducted both pre- and postoperatively.

To date, there is no standard treatment for pneumolabyrinth after CI surgery. Depending on the symptoms, cause and extent of air, a conservative approach with vertigo exercises and physiotherapy or surgical exploration with re-sealing can be discussed.

A limitation of this study is the small cohort size. Additionally, the cohort is not particularly homogeneous, as it includes one patient with a radical cavity, one patient with EVA, one patient with incomplete partition II and two others with an intralabyrinthine schwannoma. Nevertheless, to the best of our knowledge, this is the first study to describe intravestibular air in CI patients with postoperative vertigo.

In summary, there are various risk factors for postoperative dizziness with pneumolabyrinth. These risk factors include intralabyrinthine and intracranial pressure changes, e.g., due to EVA or the presence of a VP shunt. Other risk factors that favor a pneumolabyrinth are large CS or a second CS in intralabyrinthine schwannomas and an electrode location in the SV or a dislocation of the electrode from the ST to the SV.

## Data Availability

The raw data supporting the conclusions of this article will be made available by the authors, without undue reservation.

## References

[1] Basta D Todt I Goepel F Ernst A. Loss of saccular function after cochlear implantation: the diagnostic impact of intracochlear electrically elicited vestibular evoked myogenic potentials. Audiol Neurootol. (2008) 13:187–92. doi: 10.1159/000113509, PMID: 18212494

[2] Brey R Facer GW Trine MB Lynn SG Peterson AM Suman VJ. Vestibular effects associated with implantation of a multiple channel cochlear prosthesis. Am J Otol. (1995) 16:424–30. PMID: 8588641

[3] Fina M Skinner M Goebel JA Piccirillo JF Neely JG Black O. Vestibular dysfunction after cochlear implantation. Otol Neurotol. (2003) 24:234–42; discussion 242. doi: 10.1097/00129492-200303000-00018 12621338

[4] Huygen PL van den Broek P Spies TH Mens LH Admiraal RJ. Does intracochlear implantation jeopardize vestibular function? Ann Otol Rhinol Laryngol. (1994) 103:609–14. doi: 10.1177/000348949410300805, PMID: 8060053

[5] Klenzner T Neumann M Aschendorff A Laszig R. Thermische Erregbarkeit des Vestibularorgans nach Cochlear-implantation. Laryngorhinootologie. (2004) 83:659–64. doi: 10.1055/s-2004-825678, PMID: 15476138

[6] Krause E Louza JPR Hempel J-M Wechtenbruch J Rader T Gürkov R. Effect of cochlear implantation on horizontal semicircular canal function. Eur Arch Otorrinolaringol. (2009) 266:811–7. doi: 10.1007/s00405-008-0815-5, PMID: 18807058

[7] Krause E Wechtenbruch J Rader T Gürkov R. Influence of cochlear implantation on sacculus function. Otolaryngol Head Neck Surg. (2009) 140:108–13. doi: 10.1016/j.otohns.2008.10.008, PMID: 19130972

[8] Hänsel T Gauger U Bernhard N Behzadi N Romo Ventura ME Hofmann V. Meta-analysis of subjective complaints of vertigo and vestibular tests after cochlear implantation. Laryngoscope. (2018) 128:2110–23. doi: 10.1002/lary.27071, PMID: 29314057

[9] Matin F Krueger C Avallone E Rossberg W Demyanchuk A Guenther A. Influence of the electrode Array design on incidence of Vertigo symptoms and vestibular function after Cochlear implantation. Ear Nose Throat J. (2023) 102:701–8. doi: 10.1177/01455613211022075, PMID: 34182811

[10] Adunka OF Dillon MT Adunka MC King ER Pillsbury HC Buchman CA. Cochleostomy versus round window insertions: influence on functional outcomes in electric-acoustic stimulation of the auditory system. Otol Neurotol. (2014) 35:613–8. doi: 10.1097/MAO.0000000000000269, PMID: 24557034

[11] Todt I Basta D Ernst A. Does the surgical approach in cochlear implantation influence the occurrence of postoperative vertigo? Otolaryngol Head Neck Surg. (2008) 138:8–12. doi: 10.1016/j.otohns.2007.09.003, PMID: 18164986

[12] Karimi D Mittmann P Ernst A Todt I. Surgical treatment of vertigo in cochlear implantees by electrode resealing. Acta Otolaryngol. (2017) 137:1031–4. doi: 10.1080/00016489.2017.1331045, PMID: 28541825

[13] Choi JW Lyu A-R Ryu KA Kim D Park Y-H. Detection of pneumolabyrinth after temporal bone trauma using computed tomography. Acta Otolaryngol. (2016) 136:682–6. doi: 10.3109/00016489.2016.1157266, PMID: 27007704

[14] Immordino A Lorusso F Sireci F Dispenza F. Acute pneumolabyrinth: a rare complication after cochlear implantation in a patient with obstructive sleep apnoea on CPAP therapy. BMJ Case Rep. (2023) 16:e254069. doi: 10.1136/bcr-2022-254069, PMID: 37399343 PMC10314444

[15] McGhee MA Dornhoffer JL. A case of barotrauma-induced pneumolabyrinth secondary to perilymphatic fistula. Ear Nose Throat J. (2000) 79:456–9. doi: 10.1177/014556130007900611, PMID: 10893837

[16] Yanagihara N Hyodo J Takagi D Miuchi S. A case of pneumolabyrinth induced by Eustachian tube air inflation. Otol Neurotol. (2012) 33:1408–11. doi: 10.1097/MAO.0b013e31826a50dc, PMID: 22935816

[17] Vandevoorde A Williams MT Ukkola-Pons E Daval M Ayache D. Early postoperative imaging of the labyrinth by cone beam CT after stapes surgery for Otosclerosis with correlation to Audiovestibular outcome. Otol Neurotol. (2017) 38:168–72. doi: 10.1097/MAO.0000000000001306, PMID: 28068300

[18] Botti C Castellucci A Crocetta FM Fornaciari M Giordano D Bassi C. Pneumolabyrinth: a systematic review. Eur Arch Otorrinolaringol. (2021) 278:4619–32. doi: 10.1007/s00405-021-06827-0, PMID: 33881577

[19] Hallin K Stillesjö F Sundblom J Danckwardt-Lillieström N. Pneumolabyrinth following cochlear implantation resolved after shunt adjustment. Acta Oto-Laryngologica Case Reports. (2020) 5:81–5. doi: 10.1080/23772484.2020.1838906

[20] Hempel J-M Jäger L Baumann U Krause E Rasp G. Labyrinth dysfunction 8 months after cochlear implantation: a case report. Otol Neurotol. (2004) 25:727–9. doi: 10.1097/00129492-200409000-00014, PMID: 15354003

[21] Karataş E Toplu Y Gündüz E Demir İ. Severe Vertigo after Cochlear implantation: acute Pneumolabyrinth. Balkan Med J. (2018) 35:408–9. doi: 10.4274/balkanmedj.2017.1088, PMID: 29769169 PMC6158469

[22] McKinnon BJ Watts T. Subcutaneous emphysema and pneumolabyrinth plus pneumocephalus as complications of middle ear implant and cochlear implant surgery. Ear Nose Throat J. (2013) 92:298–300. doi: 10.1177/014556131309200707 23904304

[23] Moteki H Fujinaga Y Goto T Usami S-I. Pneumolabyrinth, intracochlear and vestibular fluid loss after cochlear implantation. Auris Nasus Larynx. (2018) 45:1116–20. doi: 10.1016/j.anl.2018.03.004, PMID: 29680680

[24] Ott IO Schwab B Becker H Issing PR. Pneumolabyrinth after cochlear implantation in large vestibular aqueduct syndrome. Otol Neurotol. (2008) 29:1037–8. doi: 10.1097/mao.0b013e318164cb6b, PMID: 18828208

[25] Rother T Albrecht C Issing PR. Pneumolabyrinth after cochlear implantation in large vestibular aqueduct syndrome: a case report. Am J Otolaryngol. (2011) 32:430–2. doi: 10.1016/j.amjoto.2010.07.011, PMID: 20832907

[26] Spratt JD Salkowski LR Loukas M Turmezei T Weir J Abrahams PH. Weir & Abrahams' imaging atlas of human anatomy. Philadelphia: Elesevier (2017).

[27] Im SY Kim M-K Lee S Chung J-H Choi JW. Pneumolabyrinth as an early computed tomographic finding after Cochlear implantation and its clinical significance. Otol Neurotol. (2022) 43:e38–44. doi: 10.1097/MAO.0000000000003345, PMID: 34726876

[28] Vaz F Petrus L Martins WR Silva IMC Lima JAO Santos NMS. The effect of cochlear implant surgery on vestibular function in adults: a meta-analysis study. Front Neurol. (2022) 13:947589. doi: 10.3389/fneur.2022.947589, PMID: 36034277 PMC9402268

